# Molecular epidemiology of dengue in Malaysia: 2015–2021

**DOI:** 10.3389/fgene.2024.1368843

**Published:** 2024-05-28

**Authors:** Yu Kie Chem, Surya Pavan Yenamandra, Chee Keong Chong, Rose Nani Mudin, Ming Keong Wan, Norazimah Tajudin, Rehan Shuhada Abu Bakar, Mohd Asri Yamin, Rokiah Yahya, Chia-Chen Chang, Carmen Koo, Lee Ching Ng, Hapuarachchige Chanditha Hapuarachchi

**Affiliations:** ^1^ National Public Health Laboratory, Ministry of Health, Sungai Buloh, Malaysia; ^2^ Environmental Health Institute, National Environment Agency, Singapore, Singapore; ^3^ Disease Control Division, Ministry of Health, Putrajaya, Malaysia; ^4^ School of Biological Sciences, Nangyang Technological University, Singapore, Singapore; ^5^ Saw Swee Hock School of Public Health, National University of Singapore, Singapore, Singapore

**Keywords:** dengue, molecular epidemiology, Malaysia, genetic diversity, phylogeography. severity

## Abstract

Dengue has been one of the major public health problems in Malaysia for decades. Over 600,000 dengue cases and 1,200 associated fatalities have been reported in Malaysia from 2015 to 2021, which was 100% increase from the cumulative total of dengue cases reported during the preceding 07-year period from 2008 to 2014. However, studies that describe the molecular epidemiology of dengue in Malaysia in recent years are limited. In the present study, we describe the genetic composition and dispersal patterns of Dengue virus (DENV) by using 4,004 complete envelope gene sequences of all four serotypes (DENV-1 = 1,567, DENV-2 = 1,417, DENV-3 = 762 and DENV-4 = 258) collected across Malaysia from 2015 to 2021. The findings revealed that DENV populations in Malaysia were highly diverse, and the overall heterogeneity was maintained through repetitive turnover of genotypes. Phylogeography analyses suggested that DENV dispersal occurred through an extensive network, mainly among countries in South and East Asia and Malaysian states, as well as among different states, especially within Peninsular Malaysia. The results further suggested Selangor and Johor as major hubs of DENV emergence and spread in Malaysia.

## 1 Introduction

Dengue has long been one of the major public health problems in Malaysia. Dengue fever and dengue hemorrhagic fever were first documented in Penang, a northern state of Malaysia, in 1902 and 1962 respectively (Skae, 1902; Rudnick et al., 1965). However, the disease became noticeable since early 1970s (Lam, 1993) after the first major epidemic of severe dengue due to Dengue virus type 3 (DENV-3) in 1973 (Wallace et al., 1980; Mudin, 2015). Subsequent epidemics occurred in a cyclical pattern of approximately 5–8 years [10], mainly affecting young adults living in urbanized areas, such as Selangor, Johor and Kuala Lumpur (Mohd-Zaki et al., 2014). Dengue incidence continued to increase dramatically after 2001, largely due to the factors associated with rapid urbanization (Abubakar and Shafee, 2002). Based on 2010 estimates, Malaysia spent over US$175.7 million annually on dengue prevention and control (Packierisamy et al., 2015).

Dengue is hyperendemic in Malaysia, with the co-circulation of all four DENV serotypes (AbuBakar et al., 2022). DENV-1 was first reported in Kuala Lumpur in 1954 (Smith, 1956), followed by other serotypes. Major epidemics have mainly been due to DENV-1, -2 and -3, with DENV-4 at a relatively low intensity since 1969 (Abubakar and Shafee, 2002). None of the serotypes was clearly dominant until mid-1980s but DENV-3 became dominant in 1986, followed by DENV-1, and -2 in subsequent years (Abubakar and Shafee, 2002; Mohd-Zaki et al., 2014; Ng et al., 2015). Even though DENV-4 has historically been the least common serotype, it showed a rapid upsurge during 2021–2022 (Suppiah et al., 2023). Based on the previous studies, genotypes I and II of DENV-1 have been associated with major outbreaks in Klang valley during 1987–2004 (Teoh et al., 2013), whereas DENV-2 cosmopolitan genotype caused the epidemics during 1989–2000 (Chee and AbuBaker, 2003) and in 2013 (Ng et al., 2015).

Despite the historical presence of DENV, very few studies have narrated the molecular epidemiology of dengue in Malaysia, especially in the last decade during which cases escalated further. Understanding the temporal dynamics of DENV can provide important insights into dengue epidemiology and outbreak risk assessment (Lee et al., 2010) that are vital elements of outbreak control. In this descriptive study, we therefore analyzed the genetic, evolutionary and dispersal characteristics of DENV serotypes in Malaysia from 2015 to 2021. The findings revealed a highly heterogeneous DENV population that dispersed through an extensive network, involving important hubs of virus emergence, and spread in Malaysia.

## 2 Materials and methods

### 2.1 Sample collection

**FIGURE 1:**
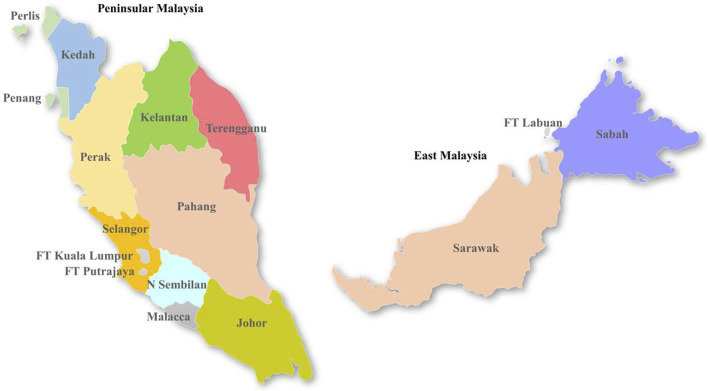
Map of Malaysia, illustrating different states and Federal Territories (FT).

Malaysia is administratively divided into 13 states and three Federal Territories, and physically separated into two regions by the South China Sea: Peninsular Malaysia and East Malaysia (Figure 1). Approximately 79% of Malaysia’s ∼30 million population lives in Peninsular Malaysia, mainly concentrated in urban areas.

All sera used to generate genome sequences in the present study were obtained through a national dengue virus surveillance program that screened in- and outpatients who sought treatment at 52 sentinel hospitals and clinics throughout the country. The clinical severity of these infections was classified into dengue fever with and without warning signs and severe dengue, according to the 2009 World Health Organization (WHO) guidelines (WHO, 2009). Severe dengue manifestations included severe plasma leakage, severe hemorrhage, and severe organ dysfunction. Serum samples obtained from suspected dengue patients were tested for dengue by using either NS1 antigen rapid test or ELISA assay. In each sentinel site, five DENV-NS1 antigen positive serum samples were randomly selected every week and were sent to the National Public Health Laboratory (NPHL) and four other regional Public Health Laboratories (PHL) for reverse transcription quantitative polymerase chain reaction (RT-qPCR) to determine DENV serotypes.

Genome sequencing was also done as part of the same dengue surveillance program. These genome sequences were shared in the online data sharing platform UNITEDengue (United in Tackling Epidemic Dengue; www.unitedengue.org), which was launched in August 2012 to support cross-border surveillance and capacity building for dengue. The analyses of shared data allow visualization of dengue incidence trend and changes in dominant serotypes and genotypes between neighboring countries. Disease Control Division of the Ministry of Health, Malaysia is a founding partner of UNITEDengue.

### 2.2 Serotyping of dengue virus

Dengue virus RNA was extracted from DENV NS1 positive sera using the NucleoSpin^®^ RNA Virus Kit (Macherey-Nagel, Düren, Germany) according to the manufacturer’s recommendations. Dengue virus serotypes were determined by using the abTES™ Kit (AITBiotech, Singapore) as per the manufacturer’s recommended protocol and guidelines.

### 2.3 Sequencing of envelope gene of dengue virus serotypes, clade classification and relatedness analysis to global sequences

Complementary DNA (cDNA) was synthesized by using extracted viral RNA and random hexamer primers, according to the protocol recommended for SuperScript III first-strand synthesis system kit (Life Technologies, Carlsbad, United States). The complete *E* gene of each serotype was amplified by using serotype-specific primers as per the protocol described elsewhere (Lee et al., 2012; Sy et al., 2023). PCR amplicons (DENV-1 = 1,780 bp; DENV-2 = 1,770 bp; DENV-3 = 1,711 bp; DENV-4 = 1,907 bp) were visualized in 1% agarose gels and were purified by using the QIAquick Gel Extraction Kit (Qiagen, Hilden, Germany) prior to sequencing at a commercial facility according to the BigDye Terminator Cycle Sequencing kit protocol (Applied Biosystems, United States). Consensus sequences for each sample were derived by assembling the overlapping raw nucleotide data in the Lasergene version 15.0 (DNASTAR Inc. Madison, WI, United States). The consensus sequences were aligned by using ClustalW implemented in BioEdit 7.2.5 software suite (Hall, 1999). The final dataset included 5,471 *E* gene sequences (4,004 complete and 1,468 partial sequences) belonging to four DENV serotypes (DENV-1 = 1,567, DENV-2 = 1,417, DENV-3 = 762 and DENV-4 = 258) reported in all states, except Labuan. This dataset was analyzed together with 17,732 *E* gene sequences (DENV-1 = 8,323, DENV-2 = 5,207, DENV-3 = 2,563 and DENV-4 = 1,639) retrieved from the GenBank database to identify distinct clades among study sequences and to determine their closest kins from other countries and thereby possible sources of their introductions by using the maximum likelihood (ML) method implemented in MEGA7 software suite (Kumar et al., 2016). Tamura-Nei model with gamma correction (TN93 + G_4_+I) was selected as the best-fit model by jModel Test for DENV-1, -2 and -3 and General Time Reversal (GTR + G_4_+I) model for DENV-4 (Darriba et al., 2012).

### 2.4 Bayesian phylogenetic and phylogeography analyses of envelope gene sequences

Bayesian time scaled phylogeny and phylogeography analyses were conducted separately for each serotype by using complete *E* gene sequences in Bayesian Evolutionary Analysis by Sampling Trees (BEAST) software package v1.10.4 (Suchard et al., 2018). The phylogenetic analyses were carried out to estimate the most probable origin, nucleotide substitution rate and the time to most recent common ancestor (tMRCA) of different DENV lineages circulated in Malaysia during the study period. The dataset, therefore included Malaysian sequences representative of all clades and those outside of any clade (non-clades). The global dataset included sequences that were interspersed within and basal to Malaysian clades and those closely related to non-clades. The global sequences were selected based on their genetic relatedness to study sequences determined by using the ML tree constructed with all study and global sequences. The final dataset used for phylogenetic analyses included 1,417 Malaysian sequences (DENV-1 = 379; DENV = 411; DENV-3 = 388; DENV-4 = 239), and 459 global sequences (DENV-1 = 147, DENV-2 = 120, DENV-3 = 119 and DENV-4 = 73).

The discrete phylogeography approach was conducted by using a symmetrical Bayesian Stochastic Search Variable Selection (BSSVS) component (Lemey et al., 2009) to understand dispersal pattern of DENV in Malaysia driven by inter-state migration and external introductions from other countries. Unlike in phylogenetic analyses, phylogeography analyses included sequences of only the most common clades that represented >90% of all sequences of each serotype (DENV-1: clades A, C and D; DENV-2: clade Ib; DENV-3: clades A, C and D; DENV-4: clades A and B) and sequences from other countries that were interspersed within each selected clade. The selected clades included 95% (n = 3,810) of 4,004 complete *E* gene sequences generated, and were therefore the main contributors of overall DENV transmission in Malaysia during the study period. The final dataset used for phylogeography analyses included 1,232 study sequences (DENV-1 = 312; DENV = 325; DENV-3 = 360; DENV-4 = 235) and 369 sequences from other countries (DENV-1 = 164; DENV-2 = 133; DENV-3 = 46; DENV-4 = 26). Malaysian sequences were categorized into 14 discrete clusters based on case locations in respective administrative states. The size of each complete dataset was trimmed by removing sequence redundancy based on each state/country and reported year to optimize computational load.

The temporal signal of final datasets for each serotype was tested by using TempEst version 1.5.3 (Rambaut et al., 2016). Tamura-Nei model with gamma correction (TN93 + G_4_+I) was used as the substitution model for DENV-1, -2 and -3 and GTR + G_4_+I for DENV-4 (Darriba et al., 2012). In order not to assume any particular demographic scenario as *a priori*, a relaxed uncorrelated lognormal clock and the Bayesian skyline plot (10-steps) coalescent model (Lemey et al., 2009) were used. The MCMC chains were run for 100 million generations sampling every 10,000 states. The output log files were visualized in Tracer v.1.5 (Rambaut et al., 2018), and Effective Sampling Size (ESS) of >200 was considered as a sufficient level of convergence of parameters. The maximum clade credibility tree (MCC) was constructed after removing the first 10% of all trees (burn-in) by using TreeAnnotator v.1.7.4. The MCC tree was visualized in FigTree v.1.4.3 (http://tree.bio.ed.ac.uk/software/figtree/). Phylogeographic reconstructions and visualization of diffusion links were conducted in SpreaD3 0.9.6 (Bielejec et al., 2016). In SpreaD3 analyses, all sequences from a particular state (discrete trait) were assigned a common latitude and longitude value that represents the center of respective state. Significant migration links between discrete traits were determined by using Bayes factor (BF) values. BF > 3 was considered well-supported, with sub-classifications of substantial (BF > 3), strong (BF > 10), very strong (BF > 30) and decisive (BF > 100) support (Faria et al., 2013). The root state posterior probability values for each discrete trait were extracted from the annotated MCC trees obtained from phylogeography analyses to determine the most probable location of origin of different virus lineages.

### 2.5 Statistical analysis

The correlation between yearly dengue cases and the proportion of serotypes in each year from 2015 to 2021 was determined by performing univariate linear regression between transformed dengue cases (centered to mean) and the proportion of serotype each year. Moreover, the association between the genotype and severity of infection was determined by using a logistic regression model with quasibinomial error structure to account for overdispersion. The binary response variable was severe (=1) or mild (=0) infection. The predictor included genotype (DENV-1 genotype I as reference due to the most common genotype) while controlling for age, and sex (binary) of a patient. All analyses were done using R version 4.3.1 (RCoreTeam, 2018).

## 3 Results

### 3.1 Dengue cases were mainly concentrated in Klang valley and Johor state during the study period from 2015 to 2021

**FIGURE 2:**
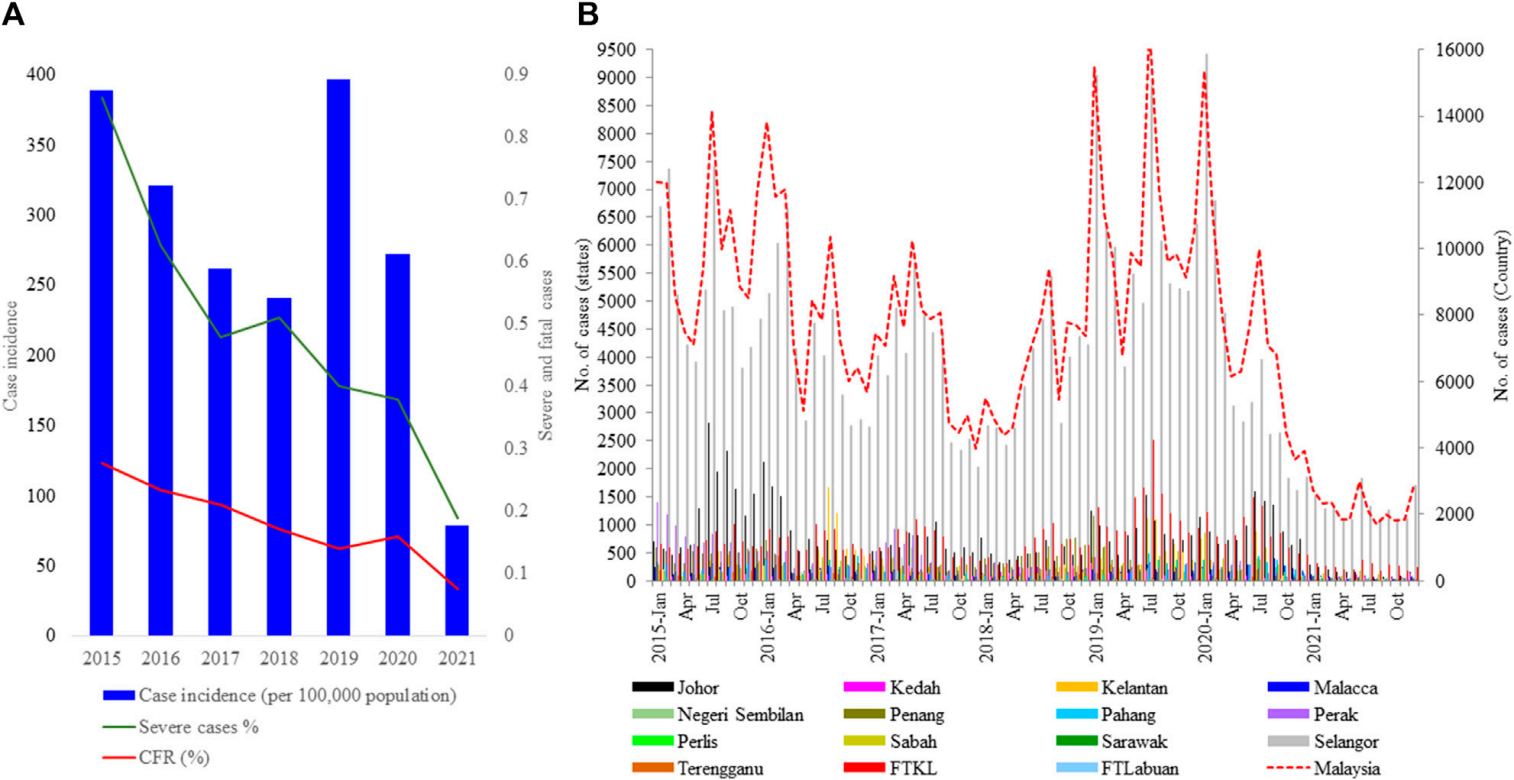
Dengue case burden in Malaysia from 2015 to 2021. **(A)**. Case incidence and percentage of severe infections and fatalities **(B)**. Distribution of dengue cases in different states. FTKL = Federal Territory Kuala Lumpur; FTLabuan = Federal Territory Labuan; FT Putrajaya = Federal Territory Putrajaya; N Sembilan = Negeri Sembilan. Population data for the calculation of annual incidence was obtained from https://data.worldbank.org.

Malaysia reported 120,836 dengue cases (389 cases/100,000 population), inclusive of 336 mortalities in 2015. The case burden trended downwards from 2016 to 2018 but escalated in 2019 to record the highest case burden (130,101 cases; 397 cases/100,000 population) reported since 2004 (Figure 2A). Even though cases trended downwards in 2020 and 2021, presumably due to COVID-19 lockdown effects (Ahmad Zaki and Xin, 2023; Iderus et al., 2023), the average annual case burden almost doubled during the period from 2015 to 2021 (90,125 cases; average incidence of 280 cases/100,000 population) as compared to the preceding 7-year period from 2008 to 2014 (∼47,000 cases; average incidence of 161 cases/100,000 population). The highest case burden during the study period was in Selangor state (53.2%), followed by Johor state (10.5%) and Federal Territory Kuala Lumpur (9.8%) (Figure 2B). Monthly cases in Selangor state closely and consistently followed the nationwide case pattern. This is not surprising because case burden has historically been high in these regions (Hassan et al., 2012) where highly urbanized and populated cities such as Klang, Petaling Jaya, Shah Alam, Kajang and Subang as well as Federal Territories Kuala Lumpur and Putrajaya are located (Figure 1). The case fatality rate (CFR) averaged at 0.2% during the 2015–2021 period and was strongly and positively correlated with dengue case numbers (Pearson’s r = 0.82, *p* = 0.02). Of 3,331 severe dengue infections (inclusive of 1,244 fatalities) reported in different states from 2015 to 2021, the highest proportions were recorded in Selangor (47.6%), Federal Territory Kuala Lumpur and Putrajaya (18.7%) and Johor (6.6%) states. The highest proportions among all dengue-related fatalities across Malaysia were also reported in the same states (Selangor-32.4%; Johor-15.6% and Federal Territory Kuala Lumpur and Putrajaya-8.3%) during the study period. Interestingly, the proportion of severe infections continued to drop from 2015 to 2021, despite having the highest case burden in 2019 (Figures 2A,B).

### 3.2 Dengue was hyperendemic, but the distribution of common serotypes varied spatially and temporally during the study period

**FIGURE 3:**
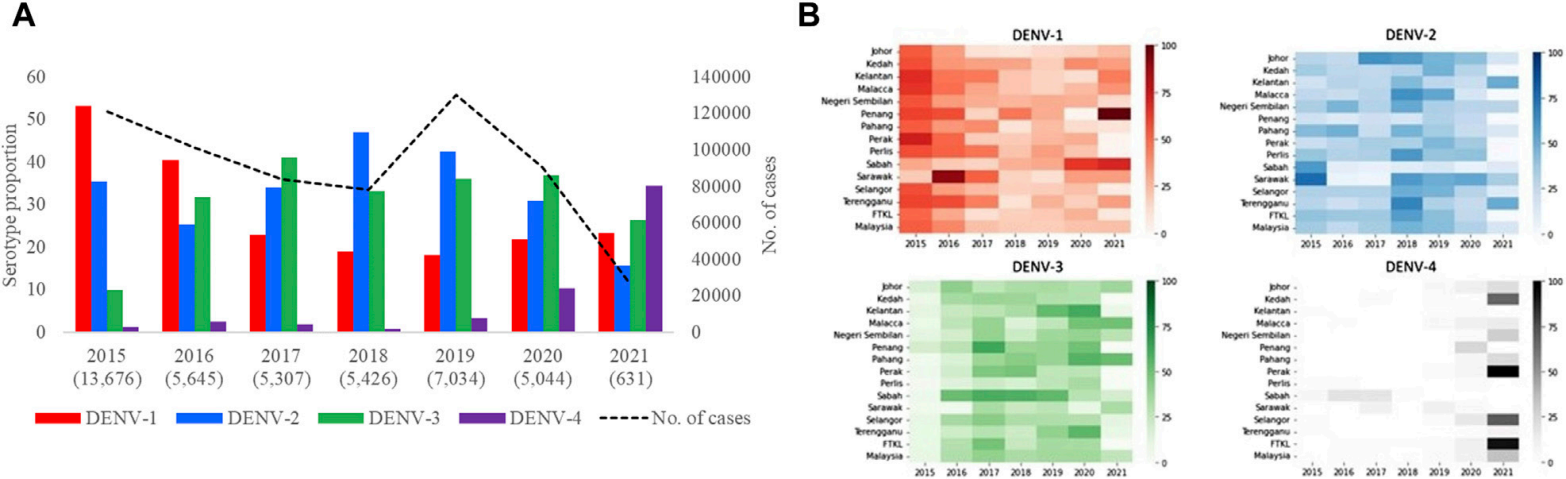
Distribution of DENV serotypes in Malaysia from 2015 to 2021. **(A)**. Temporal fluctuation of DENV serotypes in Malaysia (overall) and **(B)**. different states of Malaysia in each year. The fluctuations of all serotypes in each state are given separately in Supplementary Figure S5. Numbers within brackets in Figure 3A are the total number of samples subjected to serotyping. Substantially lower number of samples were serotyped in 2021 due to the low case burden. The legend in Figure 3B represents proportions of each serotype in different shades of respective colours. There was no serotype data from Federal Territory Labuan. FTKL = Federal Territory of Kuala Lumpur.

Based on the analysis of 42,763 clinical samples, all DENV serotypes circulated in Malaysia during the study period (Figure 3A). DENV-2 (n = 15,328; 35.9%) and DENV-1 (n = 14,302; 33.4%) were the most common serotypes, followed by DENV-3 (n = 11,678; 27.3%) and DENV-4 (n = 1,455; 3.4%). However, the proportion of each serotype fluctuated over time and one serotype was dominant periodically - DENV-1 in 2015 (53.1%) and 2016 (40.3%), DENV-3 in 2017 (41.1%) and 2020 (37%), DENV-2 in 2018 (47%) and 2019 (42.4%) and DENV-4 in 2021 (32%). None of the serotypes exceeded 50% of the total serotyped cases, except in 2015, indicating no clear dominance of a single serotype across Malaysia from 2016 to 2021. There was also no statistically significant correlation between the total number of cases reported and the proportion of serotypes across whole Malaysia in each year (DENV-1: Est = 2.68, SE = 3.16, *p*-value = 0.44; DENV-2: Est = 5.85, SE = 3.41, *p*-value = 0.15; DENV-3: Est = −1.06, SE = 4.33, *p*-value = 0.82; DENV-4: Est = −6.67, SE = 2.14, *p*-value = 0.03). It is important to note that the negative association between DENV-4 and yearly dengue cases is mostly driven by 2021 (after excluding 2021, Est = −1.61, SE = 8.70, *p*-value = 0.86). This lack of association between proportion of serotype and dengue cases was because the distribution of serotypes and their proportions differed among different states in each year (Figure 3B, Supplementary Figure S5). For example, DENV-1 was the most common serotype in all states in 2015, except Sabah and Sarawak, where DENV-2 was more common. Similarly, DENV-2 was the dominant serotype in many states in 2018, but DENV-1 and DENV-3 were more common in Penang and Sabah respectively. In general, the dominant serotype pattern was different from that of peninsular Malaysia in states such as Sabah, Sarawak and Penang that are physically distant from peninsular Malaysia (Figure 1). On the other hand, a single serotype often dominated in different states in each year, but such dominance did not show a statistically significant correlation with the total number of cases reported in each state in respective years (Supplementary Figure S5).

### 3.3 Heterogeneity of DENV populations fluctuated in time and space and different lineages dispersed through an extensive network across Malaysia during 2015–2021

**FIGURE 4:**
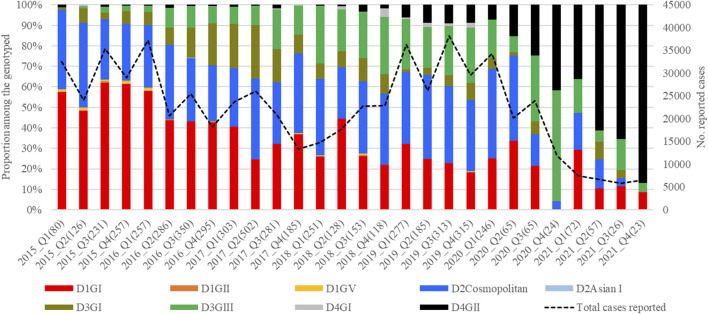
Temporal pattern of DENV genotype composition in Malaysia: 2015–2021. Each colour represents a genetically distinct genotype belonging to different serotypes. D1-D4 = DENV-1 to DENV-4; GI-GIII = Genotype I to III, Q1-Q4 = Quarter one to four.

The mean nucleotide substitution rate (subs/site/year) of each serotype did not differ substantially [DENV-1 = 7.4 × 10^−4^ (95% HPD = 6.6 × 10^-4^-8.2 × 10^−4^), DENV-2 = 9.0 × 10^−4^ (95% HPD = 8.0 × 10-^4^-9.9 × 10^−4^), DENV-3 = 8.8 × 10^−4^ (95% HPD = 7.7 × 10^-4^-9.9 × 10^−4^) and DENV-4 = 8 × 10^−4^ (95% HPD = 6.3 × 10^-4^-9.8 × 10^−4^)]. The inferred median root ages of each serotype ranged from ∼107.18 years (DENV-3) to ∼256.81 years (DENV-2), which are comparable to previous estimates (Wolf et al., 2023). Among 5,471 DENV *E* gene sequences analysed, a single genotype was largely dominant in each serotype (DENV-1 genotype I, DENV-2 cosmopolitan genotype, DENV-3 genotype III and DENV-4 genotype II) during the study period (Figure 4).

However, there were 18 distinct monophyletic clades belonging to different genotypes of each serotype, with strong posterior probability support (>0.9) (Table 1; Supplementary Figures S1-S4). Ancestral analyses (tMRCA) suggested long-term survival of all those clades (Table 1). All clades had basal sequences reported from South and East Asian countries as well as Australia (Supplementary Figures S1-S4). Each clade included sequences from many regional countries, suggesting the widespread presence of respective clades in South and East Asia and indicating the potential sources of their introduction into Malaysia (Supplementary Table S1). Even though 98.8% (1945/1969) of DENV-1 sequences belonged to genotype I, it included four distinct clades that were likely to have co-circulated, demonstrating a relatively higher heterogeneity in DENV-1 than in other serotypes. Any one of those clades dominated at a time, suggesting a consistent lineage turnover within DENV-1 (Supplementary Figure S6). Notably, DENV-1 genotype I clade D that contributed to high case burden in 2015 was replaced by clade A in Q1-2016, before a decline in the overall proportion of DENV-1 by mid-2016. This replacement coincided with an overall reduction in the total number of cases reported from 2016 to 2018, during which DENV-2 and DENV-3 gradually expanded (Figure 3A), before cases re-escalated in 2019. Only a single genotype of DENV-2 (DENV-2 cosmopolitan genotype among 99.9% (1822/1823) of DENV-2 study sequences) and two genotypes of DENV-3 (Genotype I; 41.9%; 577/1,327 and genotype III; 58.1%, 800/1,327) contributed to the case load, especially after 2015. DENV-3 genotype I was more common until the Q2-2017 than genotype III, which became more abundant subsequently, and contributed to the escalation of cases in 2019. DENV-2 and DENV-3 expansion after 2015 was also characterized by high intra-genotype diversity, with five genetically distinct clades of cosmopolitan genotype of DENV-2, two clades of genotype I and three clades of genotype III of DENV-3 (Supplementary Figures S2, S3). In DENV-4, genotypes I and II maintained a low profile from 2015 to end-2018 (Figure 4). In overall, genotype II was the most common DENV-4 lineage (90.4%, 273/302), which replaced genotype I in 2019 and became the most dominant genotype among all serotypes in 2021. In contrast to other serotypes, DENV-4 genotype II’s dominance in 2021 was associated with the lowest case burden during the study period.

**TABLE 1:** Time to most recent common ancestor (tMRCA) analysis of different lineages of DENV serotypes in Malaysia during 2015–2021.

Description	Median node height (95% HPD) in years	Estimated year of emergence^a^	Number of sequences
DENV-1 root ancestor	112 (93.1–136)	1909	NA
Genotype I (clade A)	16 (14.9–18.1)	2005	1,034
Genotype I (clade B)	14 (12.7–15.6)	2007	39
Genotype I (clade C)	15 (13.8–16.5)	2006	327
Genotype I (clade D)	14 (12.1–17.1)	2007	516
Genotype V (clade E)	13 (12.8–14.5)	2008	20
DENV-2 root ancestor	124 (93.8–164.2)	1897	NA
Cosmopolitan (clade 1b)	12 (10.1–13.3)	2009	1,508
Cosmopolitan (clade A)	13 (11.2–15.8)	2008	20
Cosmopolitan (clade B)	13 (10.1–15.3)	2008	22
Cosmopolitan (clade C)	18 (15.6–20.3)	2003	57
Cosmopolitan (clade D)	21 (18.5–23.8)	2000	13
DENV-3 root ancestor	88 (71.3–110.9)	1933	NA
Genotype III (clade A)	11 (9.6–11.9)	2010	484
Genotype III (clade B)	8 (6.6–9.7)	2013	13
Genotype III (clade C)	17 (14.9–18.9)	2004	50
Genotype I (clade D)	16 (13.1–20.6)	2005	257
Genotype I (clade E)	16 (13.0–19.2)	2005	19
DENV-4 root ancestor	150.0 (86.6–287.7)	1871	NA
Genotype I (clade A)	21 (14.0–28.8)	2000	25
Genotype II (clade B)	18 (14.9–21.5)	2003	213
Genotype II (clade C)	13 (9.1–18.4)	2008	19

^a^ Estimated year of emergence was calculated by subtracting the node height from 2021, which was the latest temporal data point in the analysis. HPD, highest posterior density.

**FIGURE 5:**
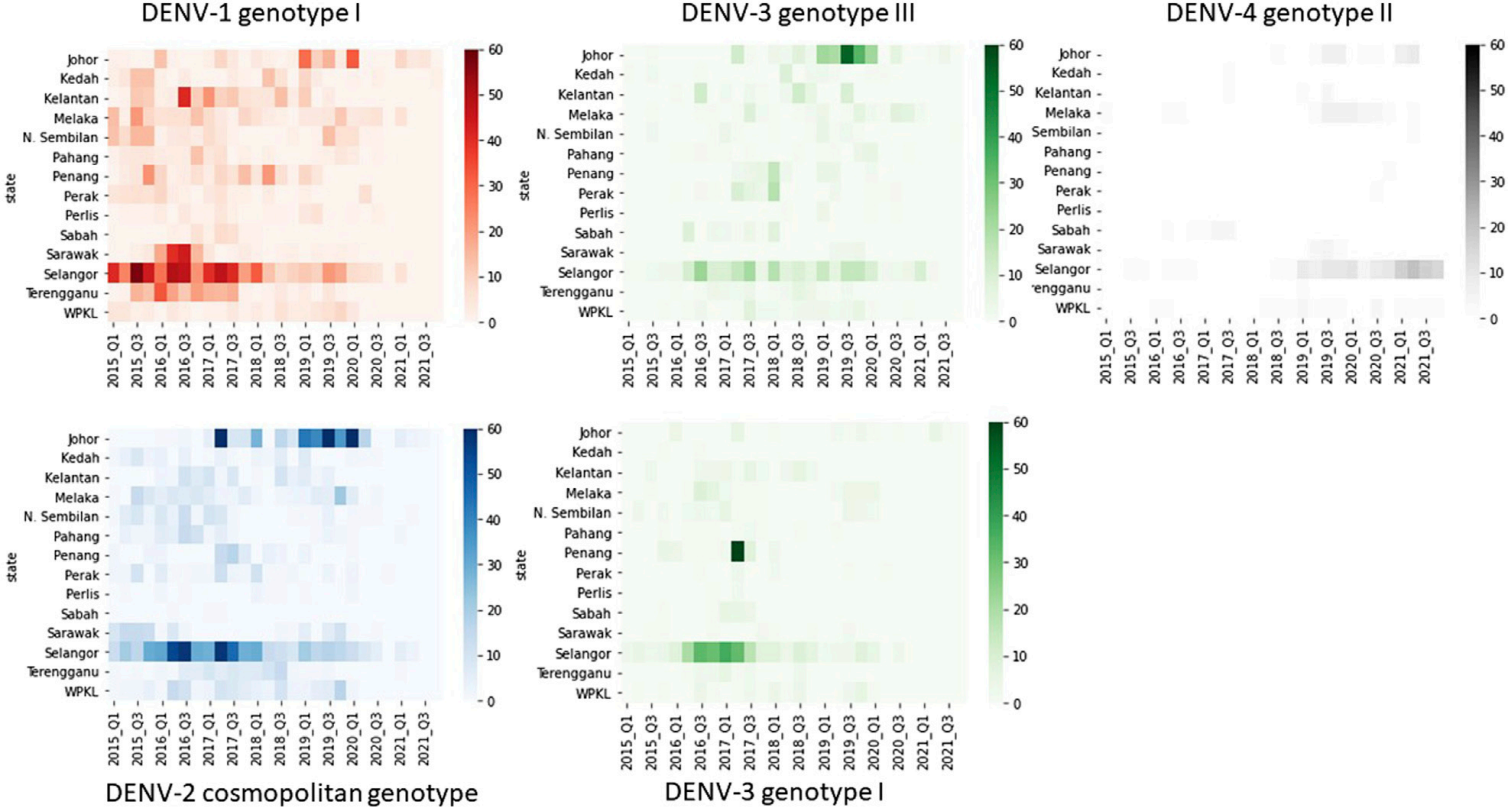
Temporal fluctuation of five most common DENV genotypes in different states from 2015 to 2021. The longitudinal pattern of DENV genotypes during the study period in different states of Malaysia was analysed by using a Python 3.6v script. Each rectangle represents data for a 3-month period. Intensity of colours indicates the number of sequences belonging to each genotype as shown in the legend. Q1-Q4 = Quarter one to four.

The distribution of common lineages also differed spatially. All common genotypes were mainly detected in Selangor and Johor states (Figure 5). Nevertheless, common genotypes such as DENV-1 genotype I, DENV-2 cosmopolitan genotype and DENV-3 genotype III were widespread throughout the study period (Figure 5). On the other hand, DENV-3 genotype I showed a notable presence in Selangor and Penang in 2016–17 but could not sustain transmission elsewhere in the country.

**FIGURE 6:**
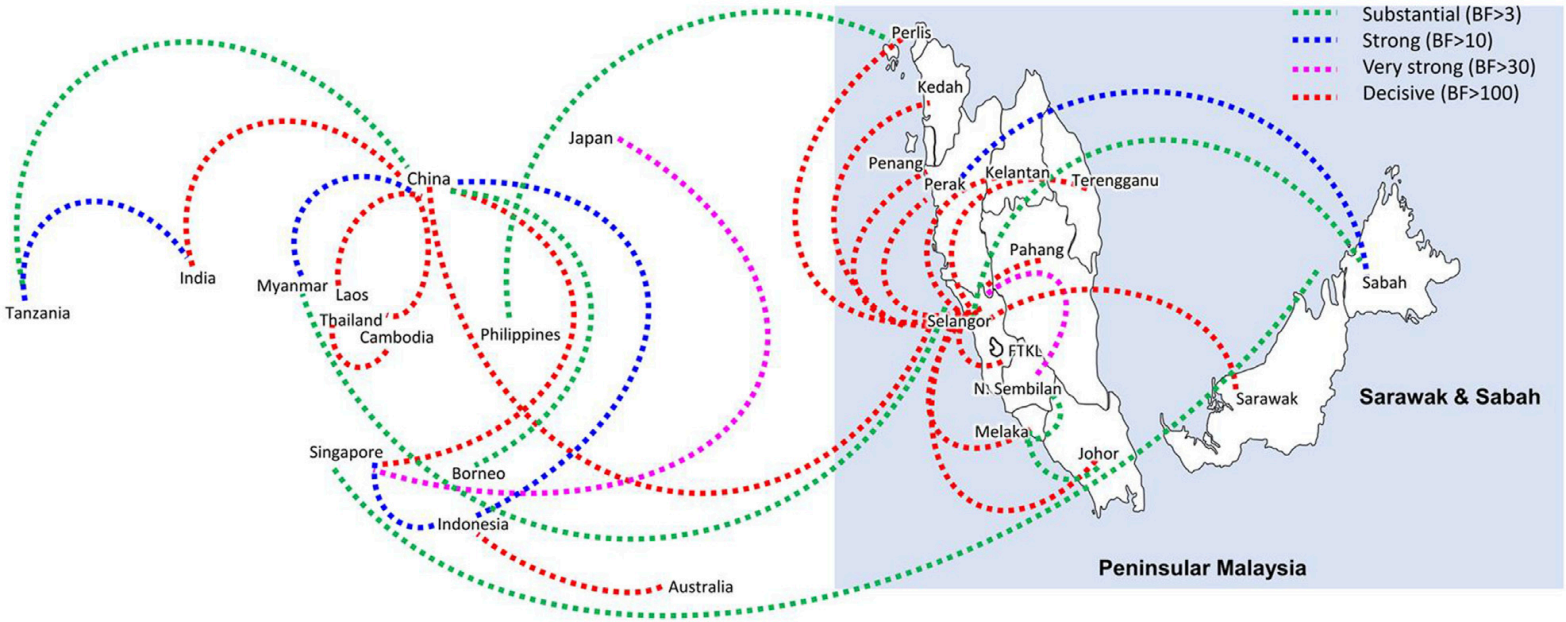
The dispersal network of common DENV-1 lineages among different countries and Malaysian states. The dispersal patterns were inferred by using the Bayesian Stochastic Search Variable Selection (BSSVS) procedure in BEAST v1.10.4 (Suchard et al., 2018). Any link supported by Bayes factor (BF) > 3 was considered as significant and only significant links are shown in the figures. The branch colour indicates the BF values as given in the legend (highest in red and lowest in green).

**FIGURE 7:**
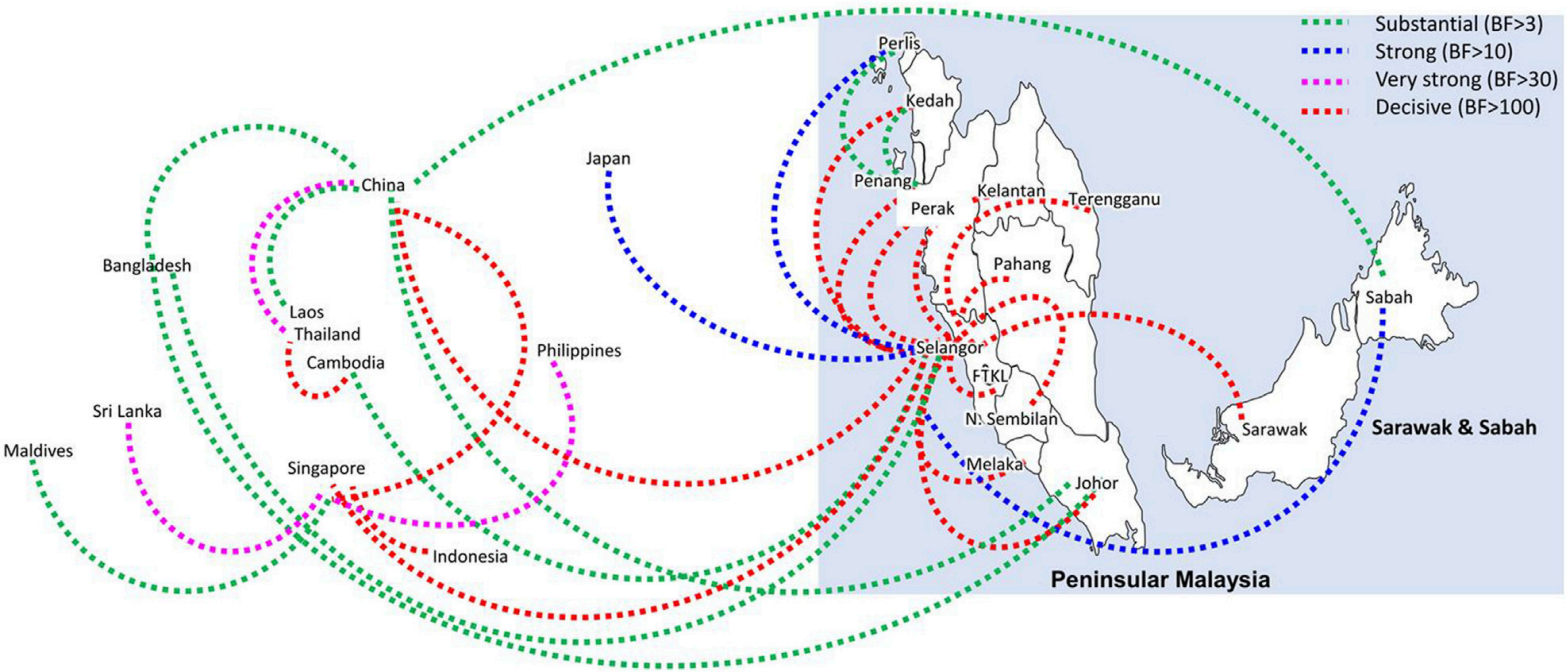
The dispersal network of the most common DENV-2 lineage among different countries and Malaysian states. The dispersal patterns were inferred by using the Bayesian Stochastic Search Variable Selection (BSSVS) procedure in BEAST v1.10.4 (Suchard et al., 2018). Any link supported by Bayes factor (BF) > 3 was considered as significant and only significant links are shown in the figures. The branch colour indicates the BF values as given in the legend (highest in red and lowest in green).

**FIGURE 8:**
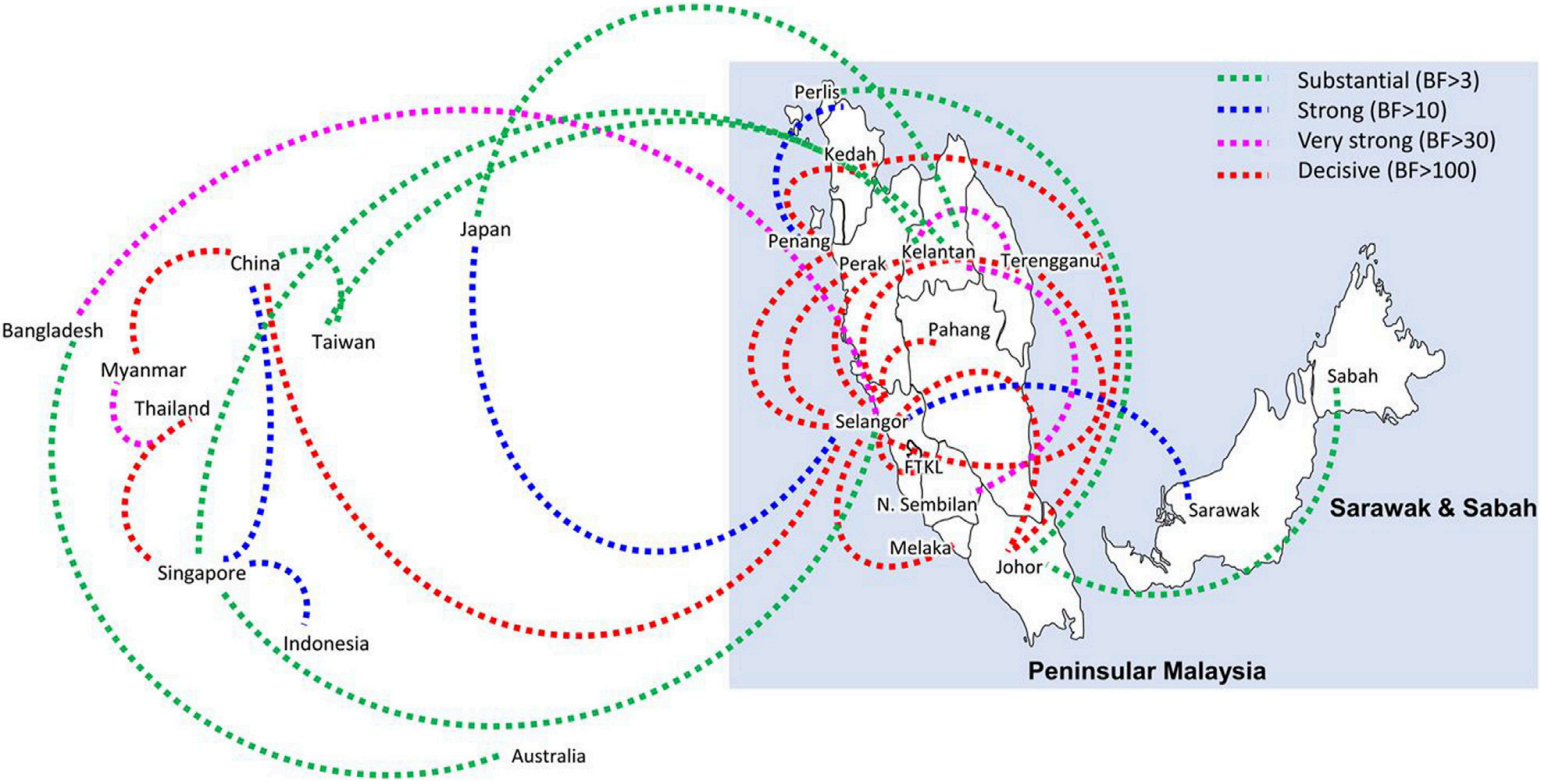
The dispersal network of common DENV-3 lineages among different countries and Malaysian states. The dispersal patterns were inferred by using the Bayesian Stochastic Search Variable Selection (BSSVS) procedure in BEAST v1.10.4 (Suchard et al., 2018). Any link supported by Bayes factor (BF) > 3 was considered as significant and only significant links are shown in the figures. The branch colour indicates the BF values as given in the legend (highest in red and lowest in green).

**FIGURE 9:**
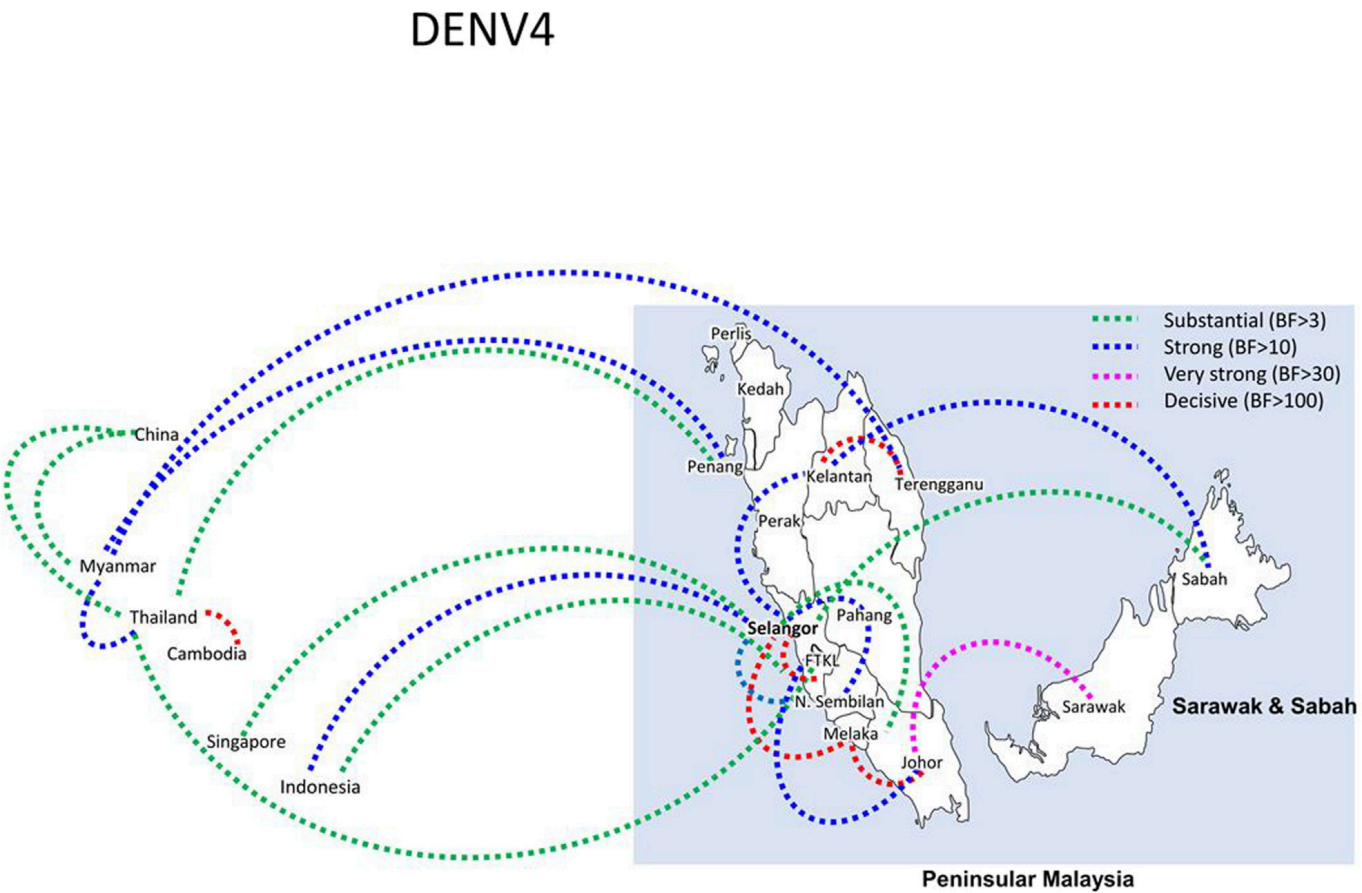
The dispersal network of common DENV-3 lineages among different countries and Malaysian states. The dispersal patterns were inferred by using the Bayesian Stochastic Search Variable Selection (BSSVS) procedure in BEAST v1.10.4 (Suchard et al., 2018). Any link supported by Bayes factor (BF) > 3 was considered as significant and only significant links are shown in the figures. The branch colour indicates the BF values as given in the legend (highest in red and lowest in green).

Bayesian phylogeography analyses demonstrated extensive dispersal of common clades of DENV serotypes between different regions, especially among countries in the South and East Asia as well as between different states in Malaysia (Figures 6-9; Supplementary Table S2). Among 25 significant links identified between Malaysian states and other countries, the highest number of external connections were with Singapore (n = 6), China (n = 4), Bangladesh (n = 3), Myanmar (n = 3) and Japan (n = 3). The highest root state probability values for each common clade showed other regional countries as the most probable ancestral location (Supplementary Table S3), suggesting that the emergence of each common clade in Malaysia was highly likely due to their importation into different states. Moreover, of 50 decisive (BF > 100) diffusion pathways identified (Supplementary Table S2), 36 connections (72%) were between Malaysian states, suggesting a strong and wide network of DENV dispersal within Malaysia. Of them, 34 (94%) connections were within Peninsular Malaysia. Of 143 total number of connections between Malaysian states, Selangor (n = 53) and Johor (n = 13) states demonstrated the highest connectivity with other states (Table 2), highlighting the potential role of these two states as hubs of DENV emergence and spread in Malaysia. This is not surprising because Selangor is an active financial and trade hub connected to other states through a well-developed transportation network. Moreover, Selangor has consistently been the major contributor of dengue burden in the country. The high diversity of DENV in Selangor and Johor also suggested their pivotal role in DENV transmission in Malaysia.

**TABLE 2:** Number of diffusion pathways of DENV1-4 with Bayes Factor >3 for each state during 2015–2021.

State	No. of diffusion pathways			
	DENV-1	DENV-2	DENV-3	DENV-4
Johor	2	3	4	4
Kedah	1	2	2	0
Kelantan	1	1	6	3
Malacca	3	1	1	2
Negeri Sembilan	2	1	1	1
Pahang	1	1	1	0
Penang	1	3	3	2
Perak	2	1	1	0
Perlis	2	2	2	0
Sabah	3	2	1	2
Sarawak	1	1	1	1
Selangor	16	16	13	8
Terengganu	1	1	2	2
FTKL	1	3	1	4

FTKL-Federal Territory of Kuala Lumpur.

### 3.4 Proportion of severe infections correlated strongly with the proportion of each genotype within the study cohort

Among 5,471 samples genotyped, 1,662 (30.4%) infections manifested severe dengue. The proportion of severe infections among the genotyped samples was substantially higher than that among total reported cases during the study period (<1%; Figure 2A) because of the inclusion of only a subset of total infections that sought treatment at 52 sentinel hospitals and clinics throughout the country. Therefore, the sample cohort used for the genotype analysis is likely to have included a relatively higher proportion of symptomatic dengue infections (severe cases and a subset of mild infections) reported during the study period. Those severe infections included 84 fatalities. Overall, the most common genotypes of each serotype contributed the highest proportions of severe infections (Table 3). The number of fatalities also followed a similar trend, with the majority (91.7%; 77/84) being caused by DENV-1 genotype I, DENV-2 cosmopolitan genotype and DENV-3 genotype III, which were in overall the most common genotypes (Table 3). DENV-3 genotype III and DENV-4 genotype II were less likely to cause severe infections compared to DENV-1 genotype I (Table 3). Older individuals were also more likely to suffer from severe infection (Est = 0.14, SE = 0.03, *p* < 0.005), but there was no statistically significant difference between sexes (Est = 0.09, SE = 0.06, *p* = 0.162).

**TABLE 3:** The association of DENV genotypes and clinical outcome.

Serotype	Genotype	Mild dengue (n = 3,809)	Severe dengue (n = 1,578)	No. of fatalities (n = 84)	Odds ratio (95% CI)
DENV-1	GI (n = 1,945)	1,309 (67.3%)	611 (31.4%)	25 (1.3%)	Reference
	GII (n = 7)	6 (85.7%)	0	1 (14.3%)	0.31 (0.04–2.56)
	GV (n = 17)	11 (64.7%)	6 (35.3%)	0	1.09 (0.40–2.97)
DENV-2	Asian I (n = 1)	1 (100%)	0	0	NA
	Cosmopolitan (n = 1,822)	1,228 (67.4%)	555 (30.5%)	39 (2.1%)	0.97 (0.85–1.12)
DENV-3	GI (n = 577)	378 (65.5%)	193 (33.4%)	6 (1%)	1.08 (0.89–1.32)
	GIII (n = 800)	629 (78.6%)	158 (19.8%)	13 (1.6%)	0.56 (0.46–0.68)
DENV-4	GI (n = 29)	23 (79.3%)	6 (20.7%)	0	0.52 (0.21–1.28)
	GII (n = 273)	224 (82.1%)	49 (17.9%)	0	0.44 (0.32–0.61)

The percentage values represent the proportion of cases under each clinical category among all samples of respective genotypes. Odds Ratios were derived from logistic regression controlling for age and gender. GI-V, genotypes I to V.

**FIGURE 10:**
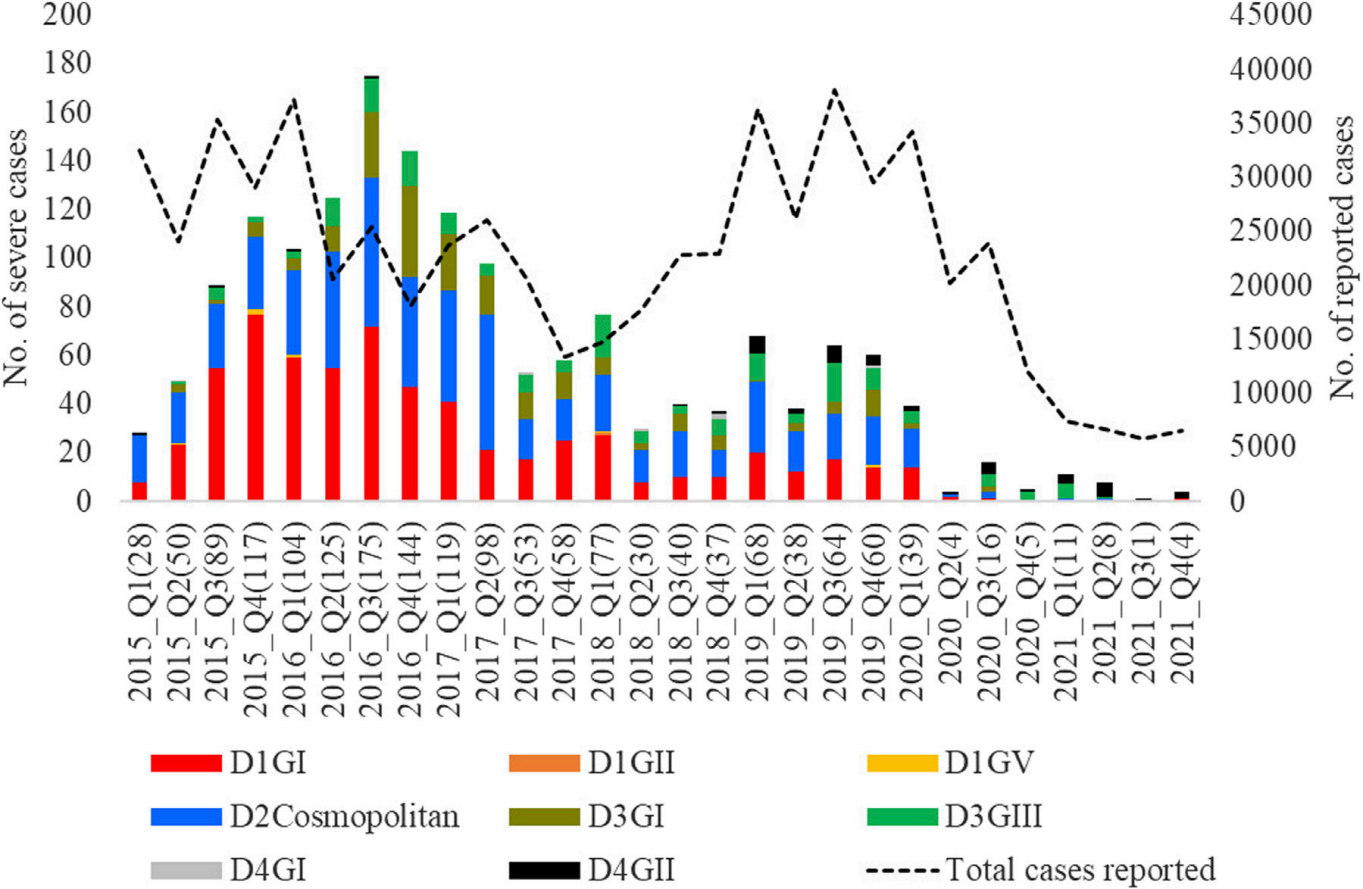
Longitudinal distribution of the number of severe infections caused by different genotypes. Severe dengue infections included cases with severe manifestations and dengue-related fatalities. FTKL- = Federal Territory of Kuala Lumpur, Q1-Q4 = Quarter one to four.

The longitudinal distribution of severe infections due to different genotypes showed that genotype I of DENV-1 and cosmopolitan genotype of DENV-2 collectively contributed the highest number of severe infections in each year from 2015 to 2020 (Figure 10). These two genotypes were the most common during the same period (Figure 4).

## 4 Discussion

Genomic surveillance forms an integral part of the pathogen surveillance and risk assessment of disease control programs. Genomic data provides valuable insights into the molecular epidemiology of pathogens of interest and thereby facilitates better understanding of the overall epidemiology and control of associated diseases (Pollett et al., 2020). This is especially applicable to fast evolving pathogens, such as RNA viruses, of which the population composition remains highly dynamic over time (Moya et al., 2000; Šimičić and Židovec-Lepej, 2022). The ensuing genetic heterogeneity is known to generate variants of high clinical significance (Šimičić and Židovec-Lepej, 2022). Genomic surveillance gained high importance during the COVID-19 pandemic, emphasizing the need of a global virus surveillance initiative (Li et al., 2022; Hill et al., 2023). Given the ability of leveraging on genome sequencing resources and technical capabilities already established in various countries, World Health Organization launched a 10-year strategy in 2022 to strengthen pathogen genomic surveillance at a global scale (Carter et al., 2022).

Dengue virus is an enveloped RNA virus transmitted by *Aedes* mosquitoes to humans (Roy and Bhattacharjee, 2021). Besides having a high mutation rate characteristic of RNA viruses, DENV’s evolution is shaped by its transmission involving a vertebrate and an invertebrate host (Lambrechts and Lequime, 2016; Yu and Cheng, 2022a). Not surprisingly, DENV populations are known to be highly heterogeneous, with constant fluctuations in the proportion of four different serotypes and various genotypes in endemic settings (Chen and Vasilakis, 2011). Empirical data has shown that turnover of DENV lineages is often associated with enhanced transmission and outbreaks (Chen et al., 2008; Lambrechts et al., 2012; Tan et al., 2022), emphasizing the importance of genetically characterizing circulating and emerging lineages in affected regions. The present study was therefore aimed at investigating the genetic, evolutionary and dispersal characteristics of DENV serotypes in Malaysia from 2015 to 2021, during which the overall dengue case burden escalated substantially (Mohd-Zaki et al., 2014; AbuBakar et al., 2022).

The findings revealed that DENV populations in Malaysia during the study period were highly diverse, and the heterogeneity was maintained across space and time through periodic fluctuations of serotypes and lineages (clades) within genotypes. This is a well-described phenomenon in major cities and countries, including Malaysia, where dengue is endemic (Weaver and Vasilakis, 2009; Lee et al., 2010; Rajarethinam et al., 2018; Harapan et al., 2020; AbuBakar et al., 2022; Yu and Cheng, 2022b). All four serotypes co-circulated at any time, confirming the hyperendemic status of dengue in Malaysia (Mia et al., 2013; Mohd-Zaki et al., 2014). Interestingly, the mean nucleotide substitution rate (subs/site/year) of each serotype did not differ substantially and were comparable to empirical data (Chen and Vasilakis, 2011), suggesting that DENV serotypes tend to evolve at a comparable rate, despite temporal fluctuations in their abundance. Besides non-uniformity in sampling that affects the observed heterogeneity in surveillance data, apparently “fixed” rate of evolution among serotypes may also be due to the strong purifying selection acting on DENV populations (Holmes, 2003; Lequime et al., 2016) that may mask the true heterogeneity of natural populations. However, their distribution differed among different states in each year (Mohd-Zaki et al., 2014), suggesting that a single serotype was not uniformly dominant across Malaysia. This observation was further supported by the inability of a single serotype to command a clear dominance across the country during the 7-year study period, except in 2015. Previous studies have shown that dominant serotype switches are associated with dengue outbreaks and such events can be used as early warning to impending outbreaks (Lee et al., 2010). Our findings therefore highlight the importance of having a comprehensive surveillance network with adequate resources in large countries/territories to harness the predictability of outbreaks based on serotype fluctuations at regional level. Moreover, such a network also enhances the ability to improve the spatial resolution of DENV genomic surveillance that is instrumental in monitoring the circulating and newly emerged lineages. As shown in our findings, genetic heterogeneity of DENV demonstrated a wide spatio-temporal spectrum across Malaysia, of which the constituent lineages are likely to have different phenotypes, fitness profiles and epidemic potential. Virus lineages with virulent phenotypes are likely to inflict high hospitalization burden. The study findings did not demonstrate a clear association between the clinical severity and the total number of reported cases. The highest proportion of severe infections was caused by dominant virus lineages. However, none of the virus lineages demonstrated a significantly high risk of causing severe infections. Instead, DENV-3 genotype III and DENV-4 genotype II were shown to be less likely to cause severe infections compared to the most common virus lineage in the study cohort (DENV-1 genotype I). The findings also showed variable proportions of severe infections when different serotypes were dominant. For example, a higher proportion of severe infections was observed when DENV-1 was dominant in 2015–2016. Interestingly, the percentage of severe infections dropped during the worst dengue outbreak recorded in Malaysia in 2019, when DENV-2 and DENV-3 were dominant. Even though the actual factors contributing to observed differences in severe infection proportions are unclear, study findings highlight the important insights achievable from a surveillance framework integrating clinical and genomic surveillance data. Genetic profiling of circulating DENV populations is also useful when monitoring the efficacy of *Wolbachia-Aedes* replacement strategy that has already been implemented in selected areas in Malaysia (Nazni et al., 2019; Cheong et al., 2023), because the emergence of DENV variants that are able to replicate despite the presence of *Wolbachia* could affect the effectiveness of replacement strategy (Yen and Failloux, 2020).

Having a strong regional genomic surveillance network is further appealing because the dominant genotypes and associated common lineages were distributed through an extensive network, mainly among countries in South and East Asia and different states across Malaysia. The phylogeographic analyses showed direct dispersal of common DENV clades between other countries and Malaysian states, highlighting the importance of understanding virus importation routes into the country. These dispersal routes play a pivotal role in the dissemination of DENV lineages from network hubs to new territories. The findings showed regional countries such as Singapore, China, Bangladesh and Myanmar as the most probable sources of virus introductions and Selangor and Johor as important dissemination hubs of their further spread in Malaysia. This is not surprising because the highest number of dominant lineages were detected in those two states, and they are highly urbanized and densely populated areas with high interstate connectivity to other peninsular regions of Malaysia. Moreover, Johor is the gateway of human movement by ground routes between Malaysia and Singapore, which may be the most potential route of virus sharing between the two countries (Ng et al., 2015). In fact, the most common DENV-2 lineage (Cosmopolitan clade Ib) during the study period is also one of the common lineages circulating in Singapore since 2013 (Hapuarachchi et al., 2016), supporting the high likelihood of cross-border virus sharing. Genomic surveillance at regional level allows the early detection of newly introduced lineages, especially those that have demonstrated high epidemic behavior elsewhere, and thereby supports an evidence-based integrated approach to control dengue in large countries, such as Malaysia.

## Data Availability

https://www.ncbi.nlm.nih.gov/genbank/The datasets presented in this study can be found in online repositories. The names of the repository/repositories and accession number(s) can be found below: https://www.ncbi.nlm.nih.gov/nuccore/pp123917, under the accession numbers PP123917- PP124551.
