# Supplementary material for: Molecular epidemiology of dengue in Malaysia: 2015–2021
Source: Front Genet. 2024 May 28;15:1368843. doi: 10.3389/fgene.2024.1368843 (PMC11165242; doi:10.3389/fgene.2024.1368843)
Supplement: Supplementary file 1 [file Table1.docx]

**Supplementary Table 1. List of countries that have reported sequences belonging to major clades of DENV-1-4 detected in Malaysia during 2015-2021**

| Serotype | Genotype | Clade | country | Year of earliest report |
| --- | --- | --- | --- | --- |
| DENV-1 | GI | Clade A | Borneo | 2017 |
|  |  |  | China | 2014 |
|  |  |  | Indonesia | 2015 |
|  |  |  | Japan | 2015 |
|  |  |  | Laos | 2014 |
|  |  |  | Myanmar | 2015 |
|  |  |  | Philippines | 2018 |
|  |  |  | Singapore | 2014 |
|  |  |  | Thailand | 2014 |
|  |  | Clade C | Cambodia | 2019 |
|  |  |  | China | 2017 |
|  |  |  | Indonesia | 2019 |
|  |  |  | Japan | 2019 |
|  |  |  | Laos | 2019 |
|  |  |  | Myanmar | 2015 |
|  |  |  | Singapore | 2015 |
|  |  |  | Thailand | 2013 |
|  |  | Clade D | Australia | 2013 |
|  |  |  | China | 2014 |
|  |  |  | India | 2019 |
|  |  |  | Indonesia | 2010 |
|  |  |  | Japan | 2014 |
|  |  |  | Singapore | 2013 |
|  |  |  | Tanzania | 2019 |
|  |  |  | Thailand | 2014 |
| DENV-2 | Cosmopolitan | Clade Ib | Bangladesh | 2017 |
|  |  |  | Cambodia | 2016 |
|  |  |  | China | 2013 |
|  |  |  | India | 2021 |
|  |  |  | Indonesia | 2014 |
|  |  |  | Japan | 2015 |
|  |  |  | Laos | 2018 |
|  |  |  | Malaysia | 2013 |
|  |  |  | Maldives | 2016 |
|  |  |  | Myanmar | 2016 |
|  |  |  | Peru | 2019 |
|  |  |  | Philippines | 2014 |
|  |  |  | Singapore | 2013 |
|  |  |  | Sri Lanka | 2017 |
|  |  |  | Thailand | 2014 |
| DENV-3 | GI | Clade D | Australia | 2017 |
|  |  |  | Bangladesh | 2017 |
|  |  |  | China | 2015 |
|  |  |  | Indonesia | 2016 |
|  |  |  | Singapore | 2009 |
|  |  |  | Taiwan | 2015 |
|  | GIII | Clade A | China | 2015 |
|  |  |  | Japan | 2019 |
|  |  |  | Myanmar | 2017 |
|  |  |  | Singapore | 2015 |
|  |  |  | Thailand | 2020 |
|  |  | Clade C | Japan | 2017 |
|  |  |  | Singapore | 2013 |
| DENV-4 | GI | Clade A | Indonesia | 2012 |
|  |  |  | Singapore | 2013 |
|  | GII | Clade B | Cambodia | 2016 |
|  |  |  | China | 2015 |
|  |  |  | Myanmar | 2013 |
|  |  |  | Thailand | 2013 |

**Supplementary Table 2. Bayes Factor and posterior probability values of significant migration events of major clades of DENV serotypes in Malaysia during 2015-2021.**

| **State connections** | | **Bayes Factor*** | **Posterior Probability** |
| --- | --- | --- | --- |
| **Location 1** | **Location 2** |  |  |
| **DENV-1** | | | |
| China | Selangor | 109345 | 1 |
| China | Thailand | 109345 | 1 |
| Kedah | Selangor | 109345 | 1 |
| Kelantan | Selangor | 109345 | 1 |
| Malacca | Selangor | 109345 | 1 |
| Penang | Selangor | 109345 | 1 |
| Perak | Selangor | 109345 | 1 |
| Selangor | Singapore | 109345 | 1 |
| Selangor | Terengganu | 109345 | 1 |
| Selangor | FTKL | 109345 | 1 |
| Johor | Selangor | 36440 | 1 |
| Pahang | Selangor | 18214 | 1 |
| Perlis | Selangor | 9929 | 1 |
| Sarawak | Selangor | 1190 | 0.99 |
| Australia | Indonesia | 1020 | 0.99 |
| Cambodia | Thailand | 324 | 0.96 |
| China | Singapore | 195 | 0.94 |
| China | Laos | 174 | 0.93 |
| Japan | Singapore | 45 | 0.79 |
| Negeri Sembilan | Selangor | 31 | 0.72 |
| China | Indonesia | 19 | 0.62 |
| India | Tanzania | 19 | 0.61 |
| Indonesia | Singapore | 17 | 0.59 |
| Perak | Sabah | 15 | 0.56 |
| China | Myanmar | 13 | 0.51 |
| Sabah | Selangor | 8 | 0.41 |
| Sabah | Singapore | 8 | 0.40 |
| China | Tanzania | 8 | 0.39 |
| Johor | Malacca | 8 | 0.38 |
| China | India | 7 | 0.35 |
| Malacca | Negeri Sembilan | 7 | 0.35 |
| Borneo | China | 6 | 0.33 |
| Myanmar | Selangor | 5 | 0.29 |
| Perlis | Philippines | 4 | 0.23 |
| DENV2 | | | |
| Johor | Selangor | 109345 | 1 |
| Kedah | Selangor | 109345 | 1 |
| Kelantan | Selangor | 109345 | 1 |
| Malacca | Selangor | 109345 | 1 |
| Negeri Sembilan | Selangor | 109345 | 1 |
| Pahang | Selangor | 109345 | 1 |
| Perak | Selangor | 109345 | 1 |
| Selangor | Singapore | 109345 | 1 |
| Selangor | Terengganu | 109345 | 1 |
| Selangor | FTKL | 109345 | 1 |
| Sarawak | Selangor | 54666 | 1 |
| China | Singapore | 4959 | 1 |
| China | Selangor | 1507 | 1 |
| Indonesia | Singapore | 278 | 0.96 |
| Penang | Selangor | 200 | 0.94 |
| China | Thailand | 71 | 0.85 |
| Philippines | Singapore | 61 | 0.83 |
| Singapore | Sri Lanka | 46 | 0.79 |
| Sabah | FTKL | 22 | 0.64 |
| Perlis | Selangor | 19 | 0.61 |
| Japan | Selangor | 12 | 0.49 |
| Kedah | Penang | 6 | 0.31 |
| Bangladesh | China | 5 | 0.31 |
| China | Johor | 5 | 0.30 |
| Bangladesh | Selangor | 5 | 0.30 |
| Penang | Perlis | 5 | 0.27 |
| China | Laos | 4 | 0.27 |
| Bangladesh | Johor | 4 | 0.27 |
| Maldives | Singapore | 4 | 0.26 |
| Cambodia | FTKL | 4 | 0.24 |
| China | Sabah | 3 | 0.21 |
| DENV3 | | | |
| China | Selangor | 91339 | 1 |
| Johor | Selangor | 91339 | 1 |
| Kelantan | Selangor | 91339 | 1 |
| Malacca | Selangor | 91339 | 1 |
| Penang | Selangor | 91339 | 1 |
| Perak | Selangor | 91339 | 1 |
| Selangor | FTKL | 91339 | 1 |
| Kedah | Penang | 30439 | 1 |
| China | Myanmar | 10140 | 1 |
| Selangor | Terengganu | 2845 | 1 |
| Pahang | Selangor | 396 | 0.98 |
| Johor | Kedah | 280 | 0.97 |
| Kelantan | Terengganu | 71 | 0.88 |
| Bangladesh | Selangor | 50 | 0.83 |
| Kelantan | Negeri Sembilan | 43 | 0.81 |
| Myanmar | Thailand | 32 | 0.76 |
| Japan | Selangor | 15 | 0.60 |
| Sarawak | Selangor | 15 | 0.60 |
| Indonesia | Singapore | 14 | 0.58 |
| China | Singapore | 14 | 0.58 |
| Penang | Perlis | 11 | 0.51 |
| Johor | Sabah | 8 | 0.44 |
| Johor | Perlis | 6 | 0.38 |
| Australia | Bangladesh | 6 | 0.36 |
| Kelantan | Taiwan | 6 | 0.36 |
| Kelantan | Singapore | 5 | 0.35 |
| Japan | Kelantan | 5 | 0.31 |
| Selangor | Singapore | 4 | 0.26 |
| DENV4 | | | |
| Selangor | FTKL | 64324 | 1 |
| Johor | Malacca | 1083 | 0.99 |
| Malacca | Selangor | 1083 | 0.99 |
| Cambodia | Thailand | 138 | 0.95 |
| Kelantan | Terengganu | 120 | 0.94 |
| Johor | Sarawak | 61 | 0.90 |
| Myanmar | Thailand | 29 | 0.80 |
| Negeri Sembilan | Selangor | 20 | 0.73 |
| Myanmar | Penang | 19 | 0.73 |
| Kelantan | Sabah | 14 | 0.66 |
| Kelantan | Selangor | 14 | 0.66 |
| Johor | FTKL | 12 | 0.62 |
| Myanmar | Terengganu | 12 | 0.62 |
| Indonesia | Selangor | 11 | 0.61 |
| Johor | Selangor | 7 | 0.50 |
| Thailand | FTKL | 6 | 0.47 |
| Sabah | Selangor | 6 | 0.44 |
| Selangor | Singapore | 5 | 0.41 |
| Penang | Thailand | 5 | 0.40 |
| Indonesia | FTKL | 4 | 0.38 |
| China | Myanmar | 4 | 0.37 |
| China | Thailand | 4 | 0.35 |

*Bayes Factor values of more than 3 are shown. FTKL-Federal Territory Kuala Lumpur

**Supplementary Table 3.** **Most probable location of origin for major clades of DENV serotypes in Malaysia during 2015-2021.**

| **DENV clades** | **Most probable local origin** | **Root state posterior**  **probability** |
| --- | --- | --- |
| DENV-1 genotype I (clade A) | China | 0.57 |
| DENV-1 genotype I (clade C) | China | 0.55 |
| DENV-1 genotype I (clade D) | Indonesia | 0.97 |
| DENV-2 cosmopolitan (clade 1b) | Singapore | 0.90 |
| DENV-3 genotype I (clade D) | Singapore | 0.96 |
| DENV-3 genotype III (clade A) | China | 0.97 |
| DENV-3 genotype III (clade C) | Singapore | 0.64 |
| DENV-4 genotype I (clade A) | Indonesia | 0.65 |
| DENV-4 genotype II (clade B) | Thailand | 0.66 |
