# Supplementary material for: Molecular epidemiology of dengue in Malaysia: 2015–2021
Source: Front Genet. 2024 May 28;15:1368843. doi: 10.3389/fgene.2024.1368843 (PMC11165242; doi:10.3389/fgene.2024.1368843)
Supplement: Supplementary file 2 [file DataSheet1.ZIP › Supplementary Figures_R1/Supplementary Figure Legends.docx]

**Supplementary Figure Legends**

**Supplementary Figure 1: Bayesian phylogeny of DENV-1.** The phylogenetic tree illustrates monophyletic clades with strong posterior probability support. The numbers on branches indicate posterior probability values. Time scale is shown below the tree. Substitution rate (substitutions/site/year) is shown in a secondary scale above the time scale. GI=Genotype I; GV=Genotype V.

**Supplementary Figure 2: Bayesian phylogeny of DENV-2.** The phylogenetic tree illustrates monophyletic clades with strong posterior probability support. The numbers on branches indicate posterior probability values. Time scale is shown below the tree. Substitution rate (substitutions/site/year) is shown in a secondary scale above the time scale.

**Supplementary Figure 3: Bayesian phylogeny of DENV-3.** The phylogenetic tree illustrates monophyletic clades with strong posterior probability support. The numbers on branches indicate posterior probability values. Time scale is shown below the tree. Substitution rate (substitutions/site/year) is shown in a secondary scale above the time scale. GI=Genotype I; GIII=Genotype III.

**Supplementary Figure 4: Bayesian phylogeny of DENV-4.** The phylogenetic tree illustrates monophyletic clades with strong posterior probability support. The numbers on branches indicate posterior probability values. Time scale is shown below the tree. Substitution rate (substitutions/site/year) is shown in a secondary scale above the time scale. GI=Genotype I; GII=Genotype II.

**Supplementary Figure 5. Annual fluctuation of DENV serotypes in different states of Malaysia from 2015 to 2021.** There was no serotype data from Federal Territory Labuan. FTKL= Federal Territory of Kuala Lumpur.

**Supplementary Figure 6. Temporal fluctuations of DENV lineages from 2015 to 2021. Data is shown quarterly.** Numbers in brackets on the X-axis are the number of samples genotyped in each quarter.
