# Supplementary figures and images for: Molecular epidemiology of dengue in Malaysia: 2015–2021
Source: Front Genet. 2024 May 28;15:1368843. doi: 10.3389/fgene.2024.1368843 (PMC11165242; doi:10.3389/fgene.2024.1368843)

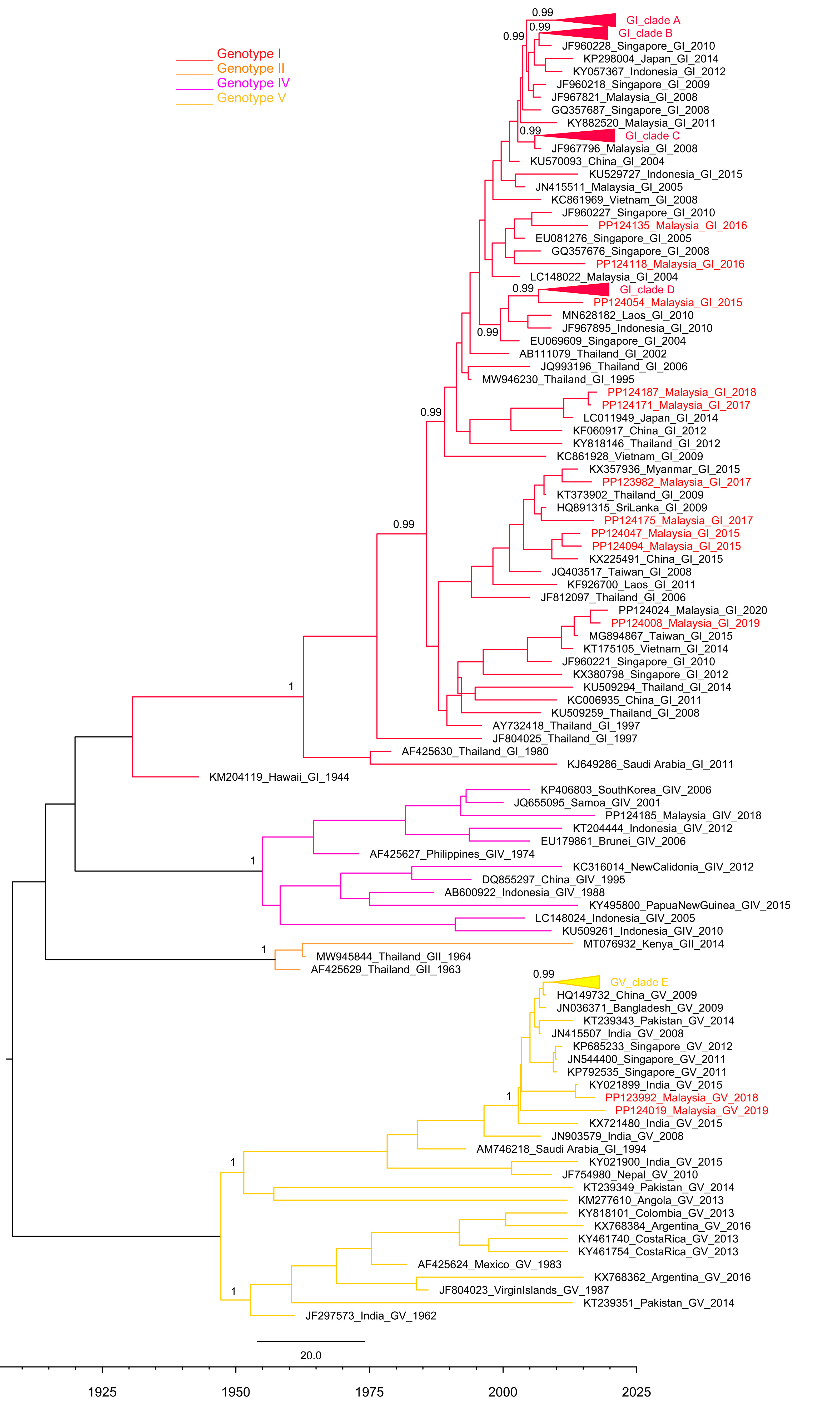

Supplement: Supplementary file 2 [file DataSheet1.ZIP › Supplementary Figures_R1/Supplementary Figure 1.jpg]

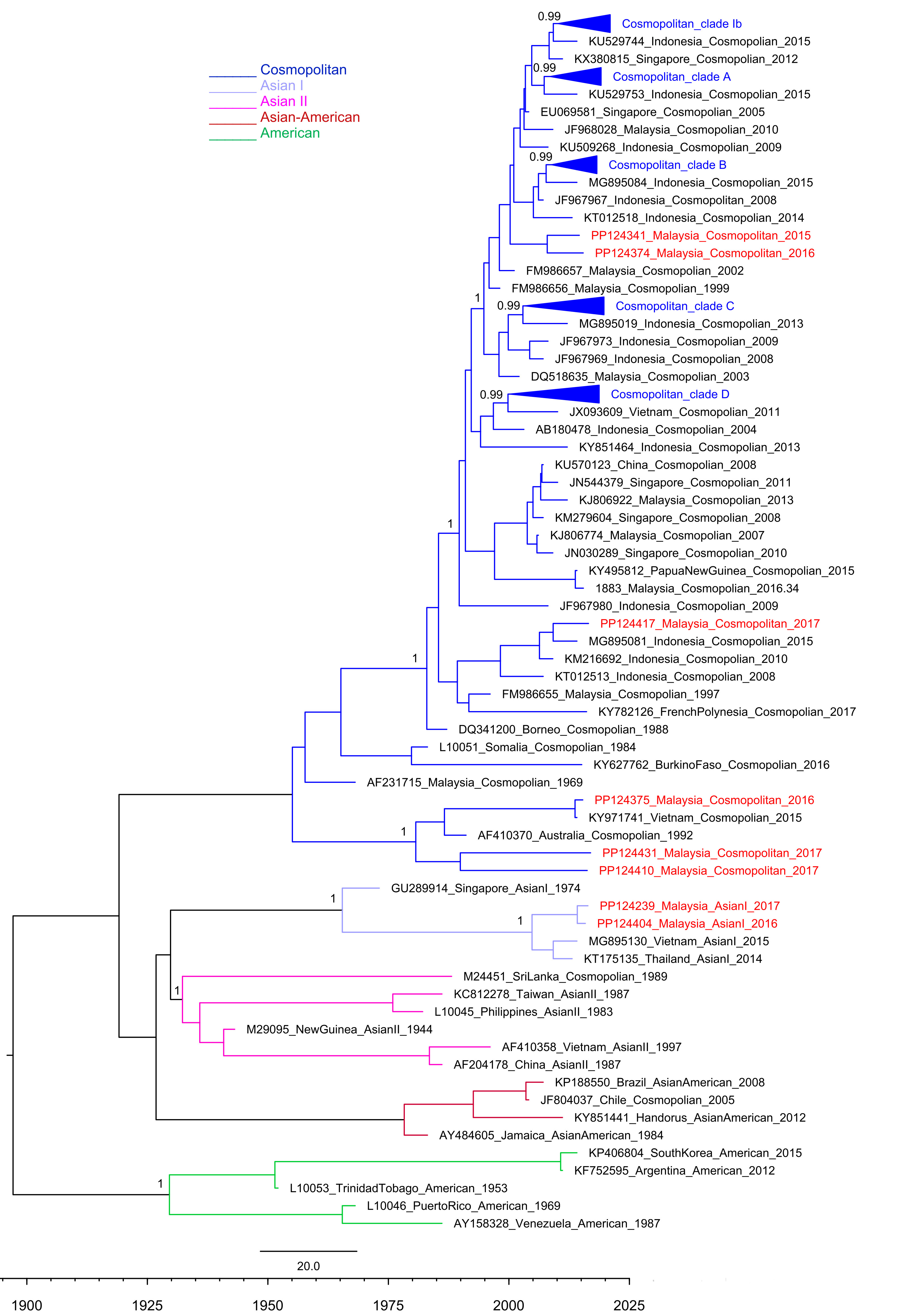

Supplement: Supplementary file 2 [file DataSheet1.ZIP › Supplementary Figures_R1/Supplementary Figure 2.jpg]

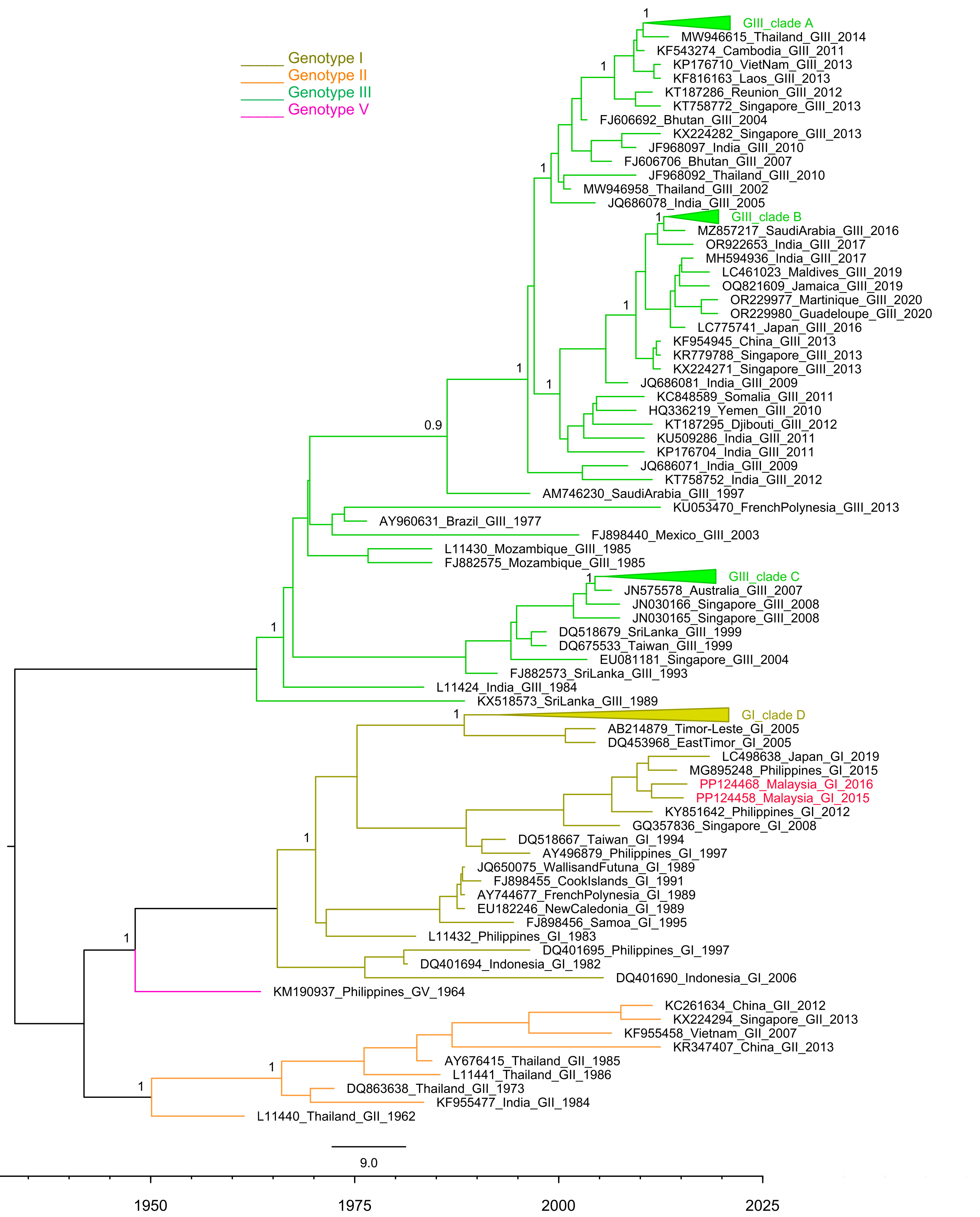

Supplement: Supplementary file 2 [file DataSheet1.ZIP › Supplementary Figures_R1/Supplementary Figure 3.jpg]

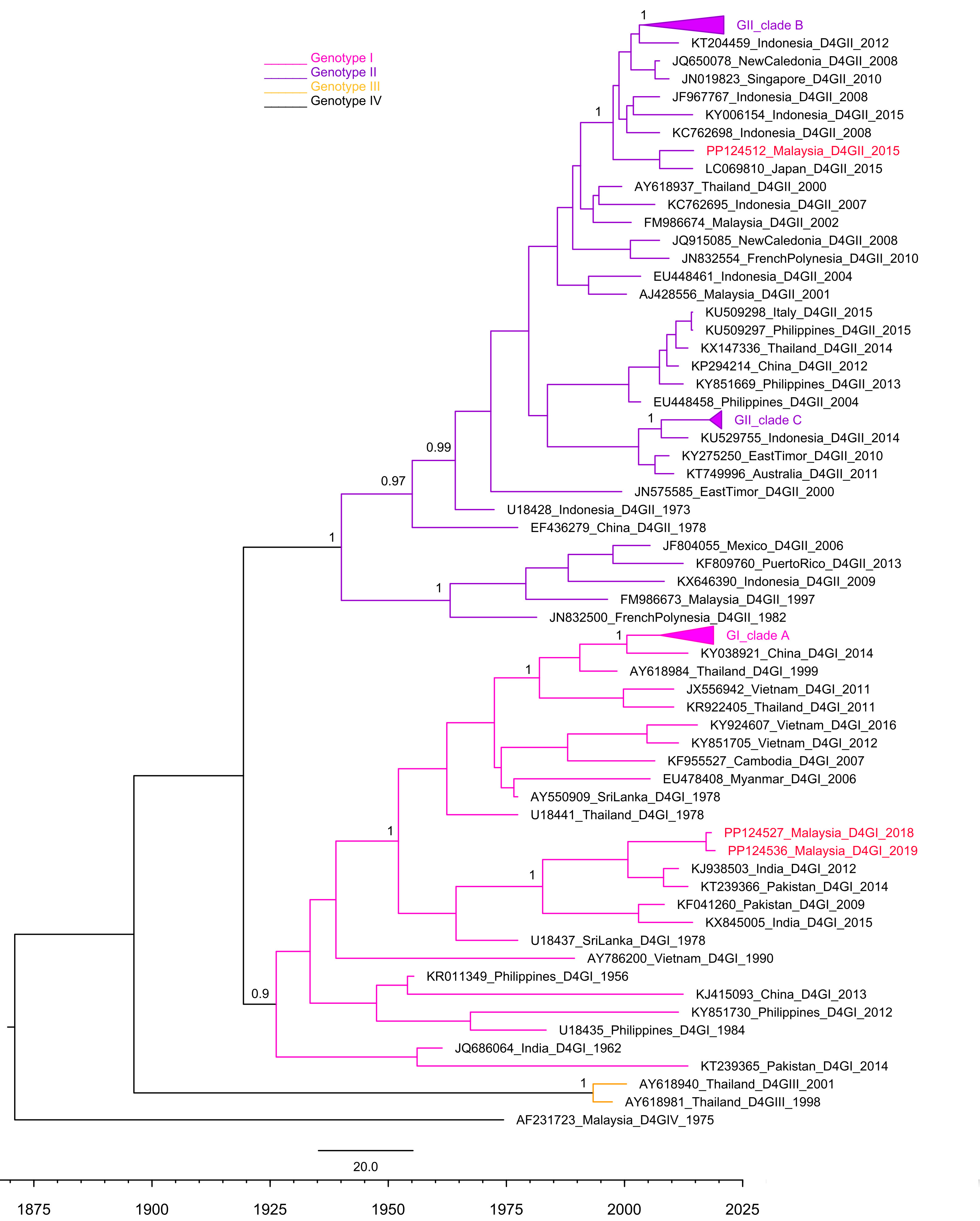

Supplement: Supplementary file 2 [file DataSheet1.ZIP › Supplementary Figures_R1/Supplementary Figure 4.jpg]

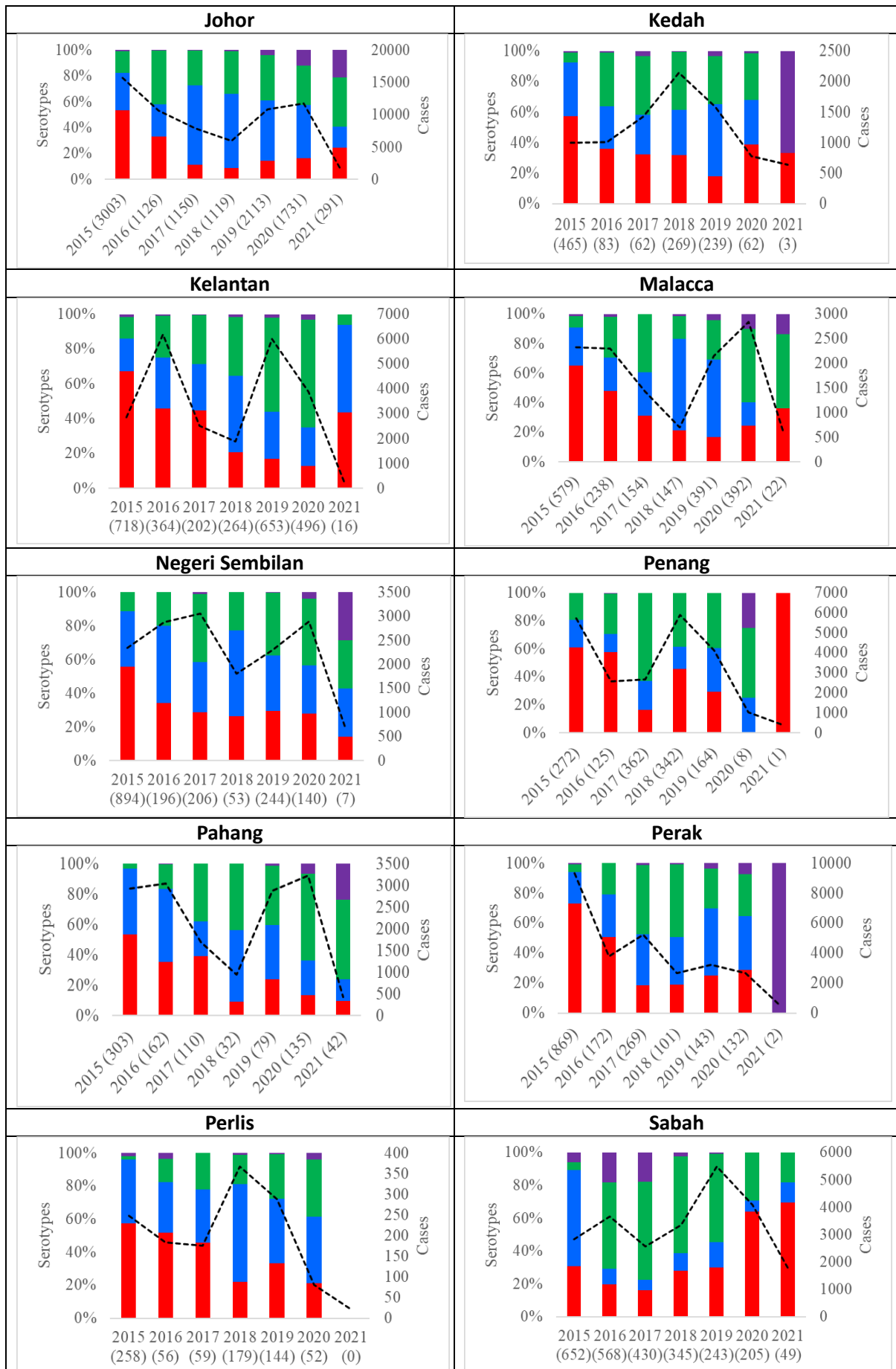

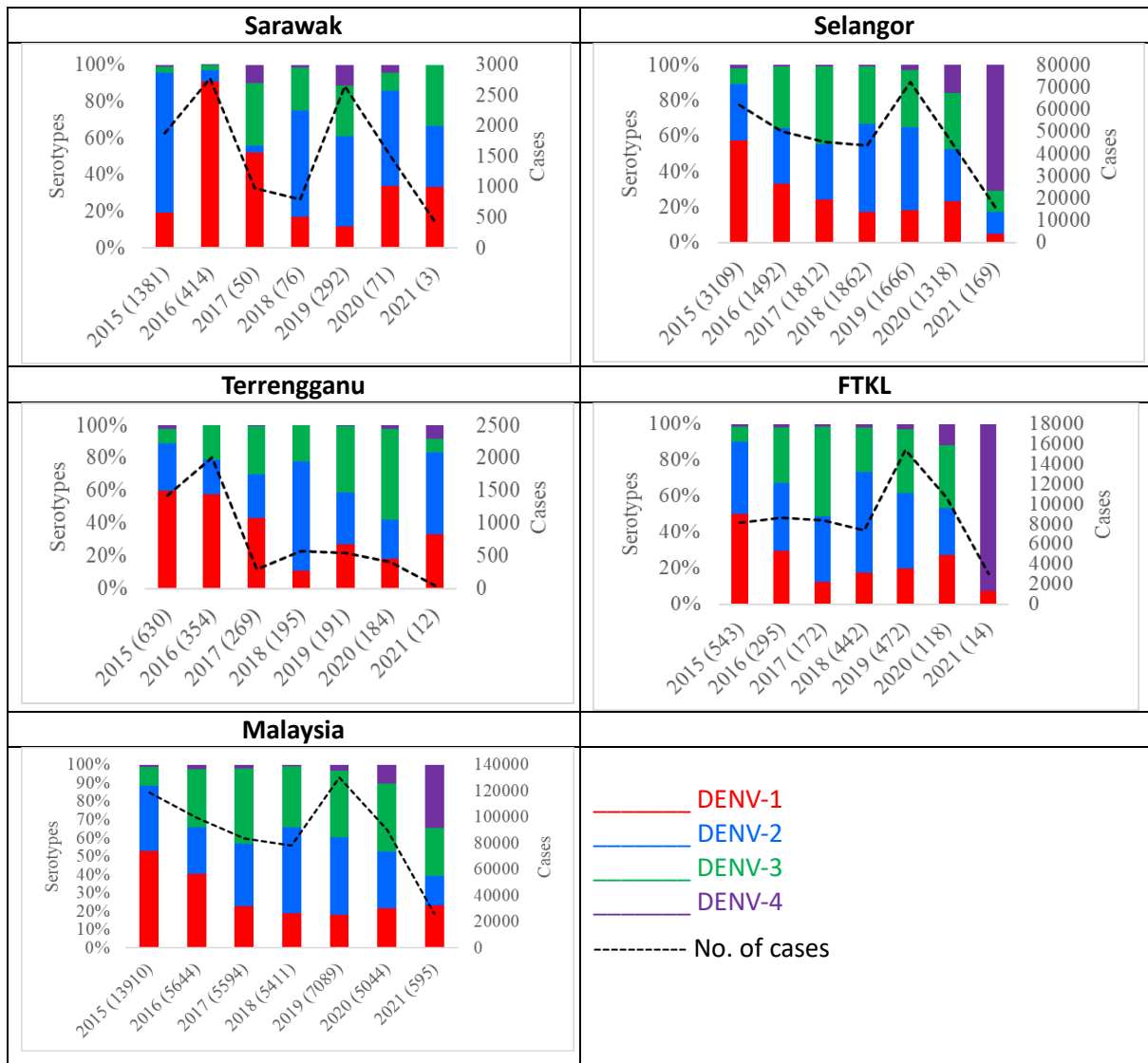

Supplement: Supplementary file 2 [file DataSheet1.ZIP › Supplementary Figures_R1/Supplementary Figure 5.pdf]

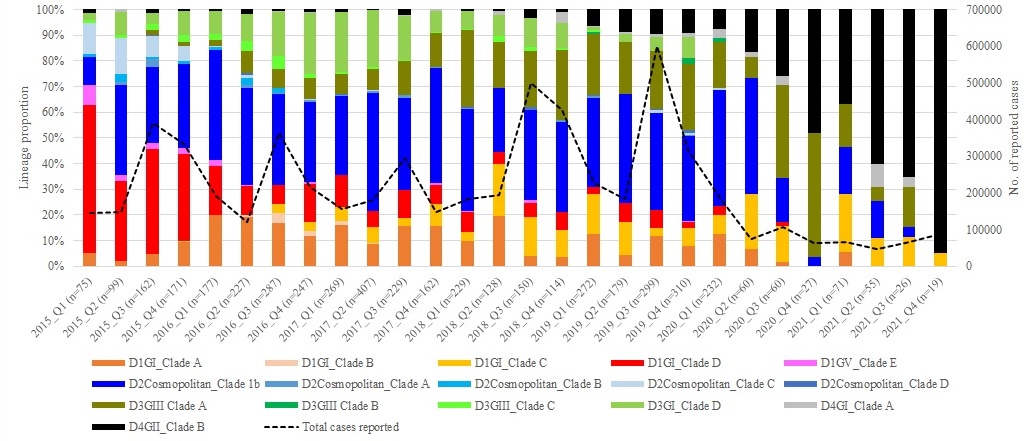

Supplement: Supplementary file 2 [file DataSheet1.ZIP › Supplementary Figures_R1/Supplementary Figure 6.jpg]
